# Biological function, mediate cell death pathway and their potential regulated mechanisms for post-mortem muscle tenderization of PARP1: A review

**DOI:** 10.3389/fnut.2022.1093939

**Published:** 2022-12-15

**Authors:** Rong Li, Ruiming Luo, Yulong Luo, Yanru Hou, Jinxia Wang, Qian Zhang, Xueyan Chen, Lijun Hu, Julong Zhou

**Affiliations:** ^1^School of Food and Wine, Ningxia University, Yinchuan, China; ^2^National R & D Center for Mutton Processing, Yinchuan, China

**Keywords:** PARP1, apoptosis and necrosis pathway, Caspase-3, tenderization, energy metabolism

## Abstract

Tenderness is a key attribute of meat quality that affects consumers’ willingness to purchase meat. Changes in the physiological environment of skeletal muscles following slaughter can disrupt the balance of redox homeostasis and may lead to cell death. Excessive accumulation of reactive oxygen species (ROS) in the myocytes causes DNA damage and activates poly ADP-ribose polymerase 1 (PARP1), which is involved in different intracellular metabolic pathways and is known to affect muscle tenderness during post-slaughter maturation. There is an urgent requirement to summarize the related research findings. Thus, this paper reviews the current research on the protein structure of PARP1 and its metabolism and activation, outlines the mechanisms underlying the function of PARP1 in regulating muscle tenderness through cysteine protease 3 (Caspase-3), oxidative stress, heat shock proteins (HSPs), and energy metabolism. In addition, we describe the mechanisms of PARP1 in apoptosis and necrosis pathways to provide a theoretical reference for enhancing the mature technology of post-mortem muscle tenderization.

## 1 Introduction

Tenderness is an important indicator to evaluate the edible value of meat. Surveys have reported that consumers have a strong desire to buy meat with better tenderness. Muscle includes muscle fibers, intermuscular fat and connective tissue (1). Its structure and complex relationship are the material basis of tenderness. Myofibrillar protein hydrolyzed by endogenous enzymes in muscle fibers is the most important factor affecting meat tenderness (2). Calpain can catalyze the degradation of myofibrils, improve the tenderness of postmortem muscles, and enhance meat quality. Huang et al. (3) used Calpain inhibitor to study its effect on chicken breast tenderness confirming that Calpain catalyzed myogenic fiber degradation is the primary reason for increased muscle tenderness and meat maturation after slaughter. During postmortem maturation, muscle triggers a series of cascade reactions due to environmental changes. Mitochondria in myocytes not only respond to ATP depletion in myocytes by accelerating the glycolytic process but also release different regulatory factors to induce apoptosis and regulate muscle tenderness. These factors include cytochrome c (cyt-c) (4), endonuclease G (Endo G), and apoptosis inducing factor (AIF) (5). At the same time, mitochondria are damaged, the body produces too much ROS, and the body is in a state of oxidative stress due to imbalance in oxygen radical metabolism in response to environmental change (6).

Poly ADP-ribose polymerase 1 is a DNA repair enzyme that has been implicated in DNA damage repair, apoptosis, necrosis, chromosome modification, and transcription (7). It can interact with Calpain and Caspase to catalyze the degradation of myogenic fibronectin, which in turn regulates muscle tenderness. However, the influence of PARP1 on meat tenderness and its related mechanism are poorly known. Therefore, this paper reviews the molecular structure characteristics and metabolic mechanism of PARP1, and summarizes the related mechanism of PARP1 in postmortem muscles participating in the pathway of apoptosis and necrosis and affecting muscle tenderness. The paper further focuses on the skeletal protein degradation and apoptosis processes to provide directions for postmortem muscle tenderization research.

## 2 PARP family and PARP1 activity

### 2.1 PARP family

Poly ADP-ribose polymerase is a family of poly ADP-ribose polymerases with 17 members identified that are largely distributed in the nucleus of eukaryotic cells, and responsible for catalyzing ADP-ribose modification in cells (8). The current family members, namely, PARP1, PARP2, and PARP3 are DNA-dependent and their common tryptophan-glycine-arginine (WGR) structural domain is a major regulator of catalytic activity, which can interact with damaged DNA to repair it in a timely manner (9). PARP1 is the most abundant and well-studied protease in the PARP family, which plays a significant role in apoptosis and DNA repair. PARP2 is responsible for base excision and single-strand break repair in DNA; it can bind specifically to damaged DNA end gaps and form a catalytic conformation. Chen et al. (10) demonstrated that PARP2 synthesizes a new poly-ADP ribose chain through the N-terminus with PARP1 located at the site of DNA damage. The N-terminal binding domain of PARP3 consists of only 40 amino acids and has a partial PARP1 N-terminal binding domain function; the WGR structural can bind to the DNA and transmits the information to the C-terminal structural domain (11). Vyas et al. (12) and Rulten et al. (13), respectively, demonstrated that PARP3 can modify the target proteins *via* the mono ADP-ribose (MAR) activity, which is different from the poly-ADP-ribose (PAR) activity of PARP1 and PARP2.

### 2.2 Structure of PARP1

**FIGURE 1:**
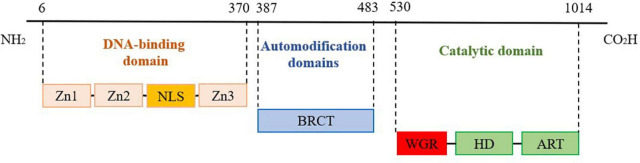
Poly ADP-ribose polymerase 1 (PARP1) structure.

Poly ADP-ribose polymerase 1, 1,014 amino acids long, consists of an N-terminal DNA-binding domain (DBD), automodification domain (AMD), and C-terminal catalytic domain (CAT), as shown in Figure 1. The DBD domain contains three zinc finger (Zn) structures, one nuclear localization signal (NLS), and an aspartate-glutamate-valine-aspartic acid (DEVD) structural related to apoptosis (14). Eustermann et al. (15) reported that Zn1 and Zn2 of the DBD domain can specifically recognize DNA damage gaps by binding to the 5′ and 3′ ends, respectively, which are distributed on both sides of DNA break sites, whereas Zn3 links the structural domains to activate the target protein. Zhou verified that NLS can recognize the Caspase cleavage site at DNA strand breaks and localize PARP1 in the nucleus. Subsequently, the two zinc finger structures can bind to and repair the DNA damage site (16). The AMD structural domain is adjacent to the WGR structural and contains a breast cancer type 1 (BRCT) structural that catalyzes PARP1-mediated synthesis of poly-ADP ribose chains. The CAT structural domain, a crucial region for linking NAD+ and catalyzing PAR synthesis, consists of the α-helical subdomain (HD) and ADP-ribosyl transferase (ART) subdomain, which contains a nicotinamide adenine dinucleotide (NAD+) binding site and a PAR catalytic site (17, 18). Rudolph et al. (19) reported that WGR, a core component of the CAT structural domain in PARP1, can interact with DNA, Zn1, Zn3, and CAT to form an inter-regional network linking the damaged DNA to the CAT structural domain. Furthermore, the Arginine (Arg) 591 site of the WGR structural domain can interact with the HD structural domain of PARP1.

### 2.3 PARP1 metabolism and activation

#### 2.3.1 PARP1 metabolism

Metabolism of intracellular PARP1 occurs *via* PAR metabolism, including the formation and degradation of PAR multimers. In the absence of DNA damage, PARP1 activity is very low. In the presence of DNA damage, PARP1 is activated and its activity increases more than 500-fold. Excessive activation of PARP1 can produce an abundance of PAR, thereby inducing the release of AIF in mitochondria after polymerization (20). Gibson and Kraus (21) suggested that PARP1 synthesized by PARP1 in the nucleus could serve as a scaffold for DNA repair and recruit DNA repair proteins to the damaged site.

**FIGURE 2:**
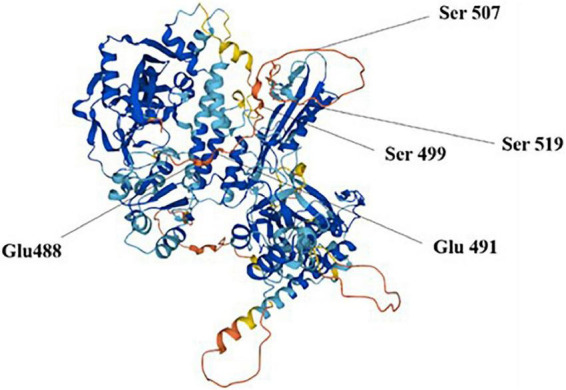
Poly ADP-ribose polymerase 1 (PARP1) modification sites.

Poly ADP-ribose polymerase 1 cleaves the substrate NAD+ to ADP ribose and niacinamide, further catalyzes ADP ribose transfer, and polymerizes to glutamate residues of nuclear proteins, thus synthesizing large homopolymer PARs with more than 200 PARs and high branching (22). Buch-Larsen et al. (23) reported that PARP1 covalently binds adenosine diphosphate-ribose units *via* lipid exchange reactions to active modification sites of glutamate (Glu) and serine (Ser) residues, including Glu 488 and 491 and Ser 499, 507, and 519, which are subsequently and repeatedly catalyzed by PARP1 to form PAR chains on PARP1 itself or target proteins (Figure 2). PAR is degraded rapidly after synthesis by poly ADP-ribose glycohydrolase (PARG), whereas the binding activity of PARP1 to DNA is reactivated after PAR degradation.

#### 2.3.2 PARP1 activation

The Zn or WGR structure in PARP1 protein binds to DNA to activate PARP1, which in turn participates in DNA repair. The activation of PARP1 is primarily caused by DNA damage. Intracellular oxidative stress can precipitate mitochondria to produce excessive reactive oxygen species (ROS, H_2_O_2_, NO, etc.) to induce DNA damage, and subsequently activate PARP1 with negative feedback. In the presence of slight DNA damage, PARP1 activation will timely repair the DNA break. In the presence of moderate damage, intracellular Caspase-3 and Caspase-7 would cleave PARP1 into PARP1-89 kDa and PARP1-24 kDa fragments to initiate cell apoptosis. However, when DNA is severely damaged, PARP1 will be overactivated and use the excess of intracellular NAD+, resulting in NAD+ depletion, reduced ATP levels, and eventually leading to cell necrosis (24). The activation of PARP1 accelerates the depletion of intracellular energy, decreases the ATP content, reduces NAD+ content, and alters the internal environment to accelerate cell death (25). In addition, the activation degree of PARP1 determines whether cells undergo apoptosis or necrosis by altering the levels of intracellular ATP (26).

## 3 PARP1 mediates cell death

Apoptosis and necrosis are the major death modes of postmortem myocytes, which are inter convertible and share similar characteristics. Cao et al. (27) observed that muscle cells of postmortem beef displayed necrosis characteristics such as vitrification of muscle fibers, lax cell nucleus and cytoplasm, and disappearance of the nucleus and cytoplasm, proving that apoptosis and necrosis of muscle cells coexisted. Similarly, Degterev et al. (28) believe that apoptosis and necrosis are two extreme types of cell death, and the two modes of death can be transformed into each other, and there will be coexistence of apoptosis and necrosis characteristics. Intracellular enzymes exist in cell apoptosis and necrosis and affect muscle tenderization by acting on cytoskeleton proteins.

### 3.1 PARP1 mediates apoptosis

**FIGURE 3:**
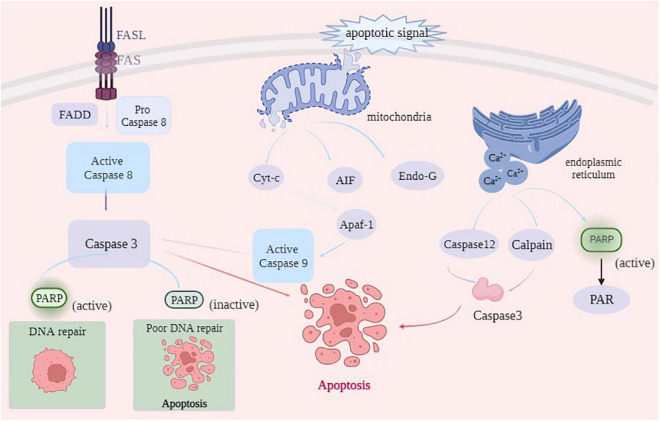
The main pathways of apoptosis.

After animal slaughter, the physiological activity of muscle tissue did not stop immediately. Under ischemia, hypoxia, and nutrient disruption apoptosis inducing factors can trigger myocytes to initiate the apoptotic program *via* coordinated control of multiple genes (29). As shown in Figure 3, the main cell-initiated apoptotic pathways include the exogenous death receptor pathway, endogenous mitochondrial pathway, PARP1 can all be involved in the onset of apoptosis through these pathways (30).

The death receptor pathway is activated by the cleavage of specific substrates by apoptotic effector enzymes. Death receptors on the cell surface bind to death ligands to recruit the Fas-associating protein with a novel death domain (FADD), which further binds to Caspase-8 precursors and activates Caspase-8 by FADD, which further binds to Caspase-8 precursor and activates Caspase-8 by forming a death-inducing signaling complex (DISC) (31). Activated Caspase-8, in turn, activates downstream Caspase-3, Caspase-6, and Caspase-7 through a cascade reaction, thus completing the apoptotic process (32). PARP1 is a substrate of Caspase, which is cleaved by Caspase-3 and Caspase-7 into PARP1 fragments (24 kDa N-terminal and 89 kDa C-terminal) in the Zn3 region, thereby losing PARP1 activity, which can retain ATP for subsequent energy consumption during apoptosis (33). Using DNA fragmentation, Lu et al. (34) demonstrated that Caspase-3 in the activated state cleaves PARP1 at specific sites into a binding structural domain (24 kDa) and a catalytic structural domain (89 kDa), leading to PARP1 inactivation. The 24 kDa PARP1 fragment is associated with DNA damage, which can bind to DNA ports and prevent the binding of intact PARP1 and DNA damage, ensuring that repair proteins are not recruited to chromosomes, resulting in DNA strand repair, and finally, complete apoptosis mediated by Caspase-3 (35). Mortusewicz et al. (36) reported that PARP1 recruited at the site of DNA damage could be cleaved, dissociating the 89 kDa fragment after cleavage and retaining the 24 kDa fragment at the site of DNA damage. The 89 kDa PARP1 fragment does not contain the DBD structural domain, and damaged DNA cannot activate it, thereby reducing intracellular energy consumption and providing sufficient energy to support apoptosis. In addition, the 89 kDa fragment catalyzes ADP ribosylation on RNA polymerase III (Pol III) complexes, activating innate immune responses and promoting apoptosis (37). Qin (38) used a high glucose-induced oxidative stress model to activate PARP1 and then inhibited PARP1 activity by ABT888. Moreover, Qin (38) confirmed that PARP1 reduced high glucose-induced cardiomyocyte apoptosis by activating the IGF-1R/Akt pathway. In conclusion, PARP1 participates in the occurrence of cell apoptosis by producing Caspase-3-mediated cleavage products of 24 kDa and 89 kDa fragments.

The mitochondrial pathway initiates in the mitochondria and is usually activated in response to injury or stress in cells within the body. When the mitochondria receive apoptotic signals, the mitochondrial membrane permeability transition pore (MPTP) is opened. Apoptotic factors, such as Cyt-c, AIF, and Endo G, are released from the mitochondria into the cytoplasm. Cyt-c released into the cytoplasm oligomerizes with apoptosis protease-activating factor (Apaf-1), leading to the conformational change in Apaf-1 to generate a heptameric apoptotic complex, which activates the apoptosis-initiating enzyme Caspase-9, thereby activating the downstream apoptosis effector enzymes Caspase-3 and Caspase-7 and initiating the apoptotic cascade response (39). However, AIF and Endo G can act directly on the nucleus, constituting another apoptotic pathway independent of apoptotic enzymes (40). Chen et al. (5) stated that gallic acid can induce apoptosis by AIF and Endo G released from the mitochondrial pathway in NCI-H292 cells. During apoptosis in the mitochondrial pathway, AIF mediates the onset of non-Caspase-dependent apoptosis by recruiting downstream nucleases that interact with cyclophilin A (Cyp A) to form active nucleases causing DNA damage by cleavage (41). After the DNA is cleaved by AIF, PARP1 functions as a protease to repair the damaged DNA and prevent chromosome shrinkage and DNA fragmentation. When large amounts of DNA are damaged, PARP1 fragments interact with AIF in the cytoplasm through the PAR polymer. PARP1 activation mediates AIF-dependent apoptosis, causing it to translocate from the mitochondria to the nucleus where apoptosis occurs. Sun et al. (42) reported that ionizing radiation can induce the death of HepG2 cells, thus releasing AIF from the mitochondria and transferring it to the nucleus by ionizing radiation, causing DNA breakage and leading to cell apoptosis. This finding indicates that ionizing radiation can induce HepG2 cell apoptosis through the AIF pathway. Yu et al. (43) demonstrated that AIF enters the mitochondria through the N-terminal mitochondrial localization signal, which is truncated by Calpain and cathepsin into a 57 kDa fragment and released into the cytoplasm in response to the death signal.

When the endoplasmic reticulum is strongly stimulated, the number of error proteins or unfolded proteins in the endoplasmic reticulum rapidly increases, beyond the range that the endoplasmic reticulum can handle, thereby disrupting the internal homeostasis and causing apoptosis (44). Calcium depletion is the primary cause of apoptosis induced by endoplasmic reticulum stress (ERS). When ERS occurs, a large amount of Ca^2+^ in the ER enters the cytosol, and activates Calpain and Caspase-12, further activating the downstream effector Caspase-3, ultimately leading to apoptosis (45). In the absence of DNA damage, the release of Ca^2+^ from the inositol triphosphate (IP3) receptor in the ER activates PARP1. Homburg et al. (46) studied nerve cells and contractile cardiomyocytes and reported that the release of intracellular stored Ca^2+^ could mediate PARP1 activation. After activation, PARP1 synthesizes excessive PAR to consume energy in cells. Caspase can timely bind to the active site of PARP1 and cleave it into fragments to destroy the PARP1 activity and provide energy for the subsequent occurrence of apoptosis.

### 3.2 PARP1 mediates necrosis

Regulatory cell necrosis is characterized by increased membrane permeability, irregular changes in the appearance of certain cells, cell membrane fragmentation, cell contents leakage, and severe inflammatory reaction (47). Cell necrosis includes programmed necrosis, iron necrosis, and cyclophilin D-dependent necrosis, Parthanatos et al. (48). Among them, Parthanatos occurs mediated by PARP1, where cells lose their integrity, phospholipid bilayer exfoliates, nucleus shrinks, mitochondrial depolarization and chromatin agglutination occurs, and the DNA is broken into fragments of approximately 50 kb, in a Caspase-independent manner.

**FIGURE 4:**
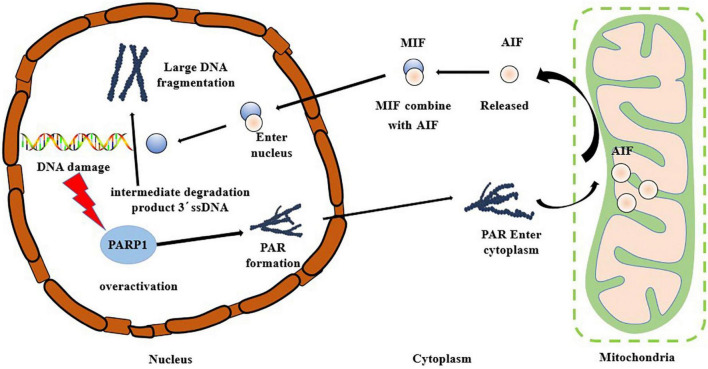
Molecular mechanism of Parthanatos.

The hallmarks of the occurrence of Parthanatos include excessive activation of PARP1, PAR accumulation, and AIF nuclear translocation. Oxidative stress can cause DNA damage, and PARP1, a DNA damage receptor, is over-activated causing the occurrence of Parthanatos (49, 50). As shown in Figure 4, PARP1 is rapidly activated after the occurrence of DNA damage, and PAR polymer accumulates in the cells and consumes a large amount of NAD+, thus inhibiting the activity of the mitochondrial oxidative respiratory chain complex enzyme, blocking the tricarboxylic acid cycle pathway, impairing mitochondrial energy metabolism, and inducing the release of AIF from the mitochondria and its transfer to the nucleus (51–53). After AIF enters the nucleus, the large DNA fragment is degraded to a 50 kb fragment. In the mode of death of Parthanatos, AIF can be combined with macrophage migration inhibitory factor (MIF) in the cytoplasm, and this complex then enters the nucleus (54). MIF can perform nuclease function to degrade 3′ ssDNA, the intermediate product in the repair process of double-strand break (DSB), leading to the failure of DNA damage repair, resulting in nuclear shrinkage, chromatin agglutination, and DNA cleavage into small fragments (43). Park et al. (55) reported that the knockout of the MIF gene protected the neurons from damage caused by necrosis, further confirming the indispensable importance of MIF in the occurrence of Parthanatos. Baritaud et al. (56) reported that AIF enters the nucleus and binds to histone H2AX and the nucleic acid endonuclease CypA, leading to the formation of DNA degradation complexes to cause chromatin agglutination and promote DNA breakage. Andrabi et al. (57) demonstrated that PARP1 fragments interact with AIF in the cytoplasm through PAR polymers. Park et al. (58) reported that β-Lapachone induced non-caspase-dependent death of hepatocytes, with significantly enhanced activity of PARP1. Simultaneously, nuclear translocation of AIF was observed. The analysis showed that β-Lapachone activated the PARP1 activity and promoted the emergence of AIF, which in turn induced hepatocyte necrosis.

## 4 PARP1 regulates the tenderization mechanism of postmortem muscles

Poly ADP-ribose polymerase 1, as a protease, can affect the structure of cytoskeleton proteins by acting on endogenous enzymes through cell apoptosis and cell necrosis, and can directly or indirectly participate in the muscle tenderization mechanism. D’Alessandro et al. (59) performed an integrated proteomics, interactomics and metabolomics analysis of Longissimus dorsi tender and tough meat samples from Chianina beef cattle. Indicating that tenderness was related to apoptosis through increase of HSPs and PARP fragment, that’s because oxidative stress promoted meat tenderness and elicited heat shock protein responses, which in turn triggered apoptosis-like cascades along with PARP fragmentation. At present, the regulatory mechanism of muscle tenderness involves Caspase, Calpain endogenous enzymes, and energy metabolism.

### 4.1 Tenderness is influenced by the regulation of cytoskeletal protein degradation

Improvement in the post-slaughter tenderness of the meat is primarily attributed to the degradation of myogenic fibrous proteins, especially structural and cytoskeletal proteins (60). Proteins highly associated with meat tenderness largely include myosin (Titin), concomitant actin (Nebulin), and troponin-T (Troponin-T) (61). At present, there are many enzymes related to the degradation of cytoskeletal proteins, including Calpain, cathepsin, and apoptotic enzymes, whose hydrolysis of cytoskeletal proteins is closely related to the improvement of postmortem muscle tenderness (62, 63).

**FIGURE 5:**
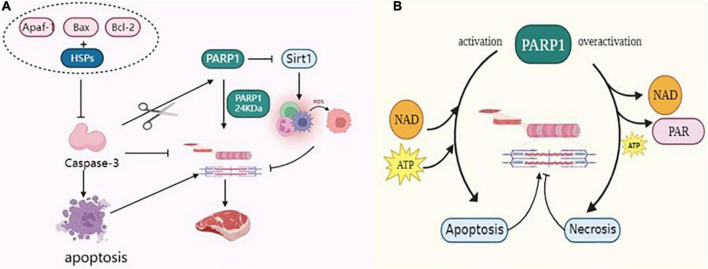
**(A)** PARP1 through Caspase-3, Sirt1, and HSPs regulates the tenderization mechanism of postmortem muscle. **(B)** PARP1 through energy metabolism regulates the tenderization mechanism of postmortem muscle.

Caspase-3 is a terminal factor in the apoptotic cascade that affects muscle tenderization by disrupting the myofibrillar structure and degrading cytoskeletal proteins. Huang et al. (64) reported that Caspase-3 inhibitors inhibited the degradation of myofibrillar proteins in skeletal muscle cells, indirectly proving that Caspase-3 was involved in the degradation of myofibrillar proteins. Activated Caspase-3, an important protease, is involved in postmortem meat tenderization and has been implicated in myofibrillar degradation during muscle maturation. Huang et al. (65) incubated myofibrillar proteins of beef skeletal muscles with recombinant Caspase-3 *in vitro* and found that numerous myofibrillar protein degradation including titin, nebulin, troponin-T etc. Indicating the involvement of Caspase-3 in protein hydrolysis in muscles. PARP1 is a marker of hydrolytic skeletal muscle protein that is specifically recognized by Caspase-3 and cleaved into 89 kDa and 24 kDa fragments. It regulates the process of the death receptor pathway in apoptosis (66, 67). Cao et al. (68) reported that caspases cleave the PARP1 of 113 kDa to 24 kDa PARP1, which is also generated by the breakdown of PARP1 substrates of caspases. Huang et al. (69) reported that Caspase-3 activation sheared PARP1 protein, accelerated the apoptosis of muscle cells in duck meat, promoted the degradation of myofibrillar proteins, and improved the tenderness of duck meat. Kemp et al. (70, 71) studied the protein hydrolysis in the skeletal muscles of pork after slaughter and reported that Caspase-3 cleaves PARP1 protein and degrades the cytoskeleton, which plays a key function in pork tenderization. Zhang (72) incubated dairy goat meat with calcium chloride and tea polyphenols during post-slaughter maturation and found that these significantly increased the protease activities of PARP1 and Caspase-3 in myocytes, resulting in increased degradation of interstitial line proteins. This finding suggested that apoptosis inducers can regulate muscle tenderness by promoting apoptosis in skeletal muscle cells (72). Saccà et al. (73) used the degradation pattern of PARP1 to assess the aging process in bovine muscles confirming that apoptotic processes occur in Longissimus lumborum (LL) and Infraspinatus (IS) during the early postmortem period and that may contribute to cell degradation of skeletal proteins. In conclusion, PARP1 is activated by caspase-3 cleavage to accelerate skeletal protein degradation (Figure 5A), thereby positively affecting the post-slaughter muscle tenderization of livestock.

Postmortem environmental changes result in excessive production of ROS in myocytes, such that the antioxidant defense system becomes insufficient to combat ROS, resulting in an imbalance between the oxidative and antioxidant systems and causing oxidative stress in the myocytes (74). Oxidative stress mediates protein oxidation, weakens the activity of proteolytic enzymes, and causes cross-linking and polymerization between protein molecules, thereby affecting the degradation of skeletal proteins, which is detrimental to the tenderization of muscles (75). Calpain degrades skeletal proteins, destroys the ultrastructure of muscle fibers, weakens the Z-line such that it disappears, causes fragmentation of myofibrils, and accelerates muscle tenderness. Carlin et al. (76) used Calpain to incubate porcine myofibrils supplemented with H_2_O_2_ and reported that oxidation changed the structure of myosin and actin and reduced the proteolytic activity of Calpain. Chen et al. (77) reported that packaging porcine dorsal longissimus muscle with high oxygen during storage enhanced the hydrophobicity of the protein surface, increased the carbonyl content, and enhanced protein oxidation. Protein oxidation inhibited the activity of μ-Calpain in the muscles, thereby delaying the degradation of skeletal proteins such as troponin-T and intercalary line proteins in pork. Silent information regulator 1 (Sirt1) enhances mitochondrial biogenesis, upregulates the activities of antioxidant enzymes superoxide dismutase (SOD) and succinate dehydrogenase (SDH) to play an antioxidant role and increases the expression of Sirt1, thus inhibiting the accumulation of ROS in myocytes (78). In contrast, excessive activation of PARP1 inhibits the expression of Sirt1, reduces the activity of mitochondrial complex I, inhibits the function of nicotinamide adenine dinucleotide (NADH) oxidase, induces the uncoupling of mitochondrial electron transport chain, induces the generation of superoxide anion radical (O_2_-^–^), thus aggravating the oxidative stress in myocytes (79, 80). Cantó et al. (81) reported that over-activation of PARP1 can deplete large amounts of NAD+, thereby reducing intracellular NAD+ content to 20–30%, which in turn inhibits Sirt1 function and leads to increased levels of intracellular oxidative stress. Zhang et al. (82) enhanced the ubiquitination of PARP1 in mouse cells and found that the oxidative stress of cells was reduced following PARP1 proteasome degradation. As shown in Figure 5A, the overexpression of PARP1 inhibited Sirt1 activity, aggravated oxidative stress in myocytes, weakened the ability of proteolytic enzymes to degrade skeletal proteins, and reduced meat tenderness. Therefore, muscle tenderness can be improved by regulating PARP1 activity during postmortem maturation.

### 4.2 Tenderness is influenced by the regulation of apoptotic processes

Apoptosis enzyme belongs to cysteine protease and is mainly involved in cell apoptosis, which plays an important role in the transformation of muscle into meat. Therefore, the process of cell apoptosis is closely related to the tenderness of meat.

Heat shock proteins (HSPs) are a class of anti-apoptotic chaperone proteins, also known as heat stress proteins, that regulate the mitochondrial apoptotic pathway and can interact with apoptotic bodies (Apaf-1, Bax, Bcl-2, etc.), thereby preventing the activation of Caspase-3 and inhibiting the degradation of interstitial line proteins, and are molecular markers of meat tenderness (83, 84). As shown in Figure 5A, HSPs can bind to Caspase-3 to reduce its activity, which in turn affects the apoptotic process of cells. Ding et al. (85) reported that HSP27 binds to Caspase-3 during beef ripening and reduces the activity of Caspase-3, thereby inhibiting the degradation of myogenic fibrous proteins and thus hindering muscle tenderization. Balan et al. (86) observed the degradation of HSP27 in post-slaughter beef and reported that its degradation significantly correlated with the degradation of interstitial line proteins and troponin-T, and hypothesized that HSP27 degradation enhanced the hydrolysis of myogenic fibronectin by endogenous enzymes. Further studies demonstrated that HSPs not only bind the myofibrillar protein to act as a substitute for μ-Calpain, competitively inhibit μ-Calpain activity, and protect the integrity of myofibrils by reducing the enzymatic hydrolysis of myofibrillar protein, but also bind to the substrate site of Caspase-3 to inhibit its activity, followed by inhibiting the degradation of skeleton proteins (87). On the contrary, PARP1 is the activation substrate of Caspase-3, which can activate Caspase-3 and subsequently cleave PARP1 such that it loses its biological activity. Activated Caspase-3 can accelerate apoptosis and promote the degradation of skeletal proteins, which ameliorates muscle tenderness. Therefore, PARP1 can compete with HSPs for the Caspase-3 substrate site, promote the activation of Caspase-3, accelerate the degradation of myofibrillar protein, and facilitate the tenderization of postmortem muscles.

The ATP content in cells can affect cell apoptosis or necrosis, which is the crucial factor determining the cell death mode. Apoptosis requires energy consumption; ATP is required for the activation of Apaf-1 and Caspase precursors generated in the mitochondrial pathway (88). When energy is exhausted, apoptosis is inhibited, energy dependent life processes in myocytes stop, cell membranes are disrupted, and cytoplasm leaks out, leading to cell necrosis. Mitochondria are central regulators of apoptosis and provide sites for metabolic pathways such as oxidative phosphorylation, tricarboxylic acid cycle, energy conversion, and calcium ion storage under normal conditions (89, 90). After animal slaughter, the disruption of communication between muscle tissue and the outside world leads to tissue ischemia and hypoxia, which then leads to excessive ROS production in the metabolic processes of muscle cells. This cascade affects the functions of skeletal muscle mitochondria and damages the DNA structure in the cells, thus activating PARP1. The level of PARP1 activity is indirectly related to the mode of cell death, thus delaying or promoting muscle tenderization. The occurrence of necrosis is related to the decrease of intracellular NAD+ concentration. Excessive activation of PARP1 decreases NAD+ concentration, which is the primary factor causing the abnormal energy metabolism of cells. Simultaneously, PARP1 consumes a large amount of ATP while generating the PAR polymer, thus turning the cell death process from apoptosis to necrosis, which is not conducive to the tenderization of meat. However, NAD+ is a cofactor of glyceraldehyde 3-phosphate dehydrogenase (GAPDH), which participates in redox reactions. In addition, its depletion prevents ATP production by glycolysis in postmortem muscles. Chen et al. (91) used an acyl-CoA-binding domain containing 3 (ACBD3) to activate PARP1 and reported that a large amount of PAR was synthesized in the cytoplasm, which, in turn, reduced the intracellular NAD+ content. The introduction of mutant genes on the DEVD structural of PARP1 generates the mutant PARP1 that cannot be cleaved. The mutant PARP1 protein loses PAR activity and does not consume intracellular ATP and NAD+ (92). As shown in Figure 5B, PARP1 consumes large amounts of NAD+ and ATP, depleting intracellular energy and leading to cell necrosis, which in turn inhibits the activity of apoptosis enzymes. Caspase affects the tenderization of postmortem muscles, and inhibition of its activity delays the degradation of myogenic fibronectin in myocytes, which is negatively correlated with meat tenderness and is detrimental to post-slaughter meat tenderization (93).

## 5 Conclusion

This review details the structure and metabolic mechanism of PARP1, discusses the mechanism by which PARP1 mediates apoptosis and necrosis, and analyzes the potential mechanism by which it regulates muscle from the perspective of its ability to be activated by affecting oxidative stress and energy metabolism, get activated by Caspase-3 cleavage, and influence the competition for substrate sites by HSPs. PARP1 as a DNA damage receptor, in the presence of moderate DNA damage, PARP1 activation and participates in apoptosis through death receptors, mitochondria, and endoplasmic reticulum. In the presence of severe DNA damage it mediates the Parthanatos cell necrosis pathway. During the conversion of livestock from muscle to meat after slaughter, the mode of cell death affects the tenderness of the meat. Apoptosis activates Caspase-3, which destroys the structural integrity of muscle fibers and improves muscle tenderness, whereas cell necrosis exacerbates the accumulation of ROS and inhibits the activity of apoptotic enzymes, which is detrimental to post-slaughter meat tenderness.

We believe that although studies on regulating meat tenderness by regulating PARP1 are scarce, PARP1 can provide novel ideas for subsequent muscle quality research. In the future, multiomics analyses such as metabolomics, transcriptomics, and proteomics can be used to study the link between PARP1 and meat tenderness. In addition, new ideas can be explored to improve meat tenderness after slaughter. The aim is to provide a theoretical reference for the development of high-quality and safe meat production technology.

## Author contributions

YL: conceptualization, project administration, and funding acquisition. RLi, YH, JW, QZ, XC, LH, and JZ: resources. RLi: writing—original draft preparation. YL and RLu: writing—review and editing. All authors read and agreed to the published version of the manuscript.
